# Molecular Dissection of Quantitative Variation in Bermudagrass Hybrids (*Cynodon dactylon* x *transvaalensis*): Morphological Traits

**DOI:** 10.1534/g3.119.400061

**Published:** 2019-06-17

**Authors:** Sameer Khanal, Jeffrey C. Dunne, Brian M. Schwartz, Changsoo Kim, Susana Milla-Lewis, Paul L. Raymer, Wayne W. Hanna, Jeevan Adhikari, Susan A. Auckland, Lisa Rainville, Andrew H. Paterson

**Affiliations:** * Plant Genome Mapping Laboratory, University of Georgia, Athens, GA 30606,; † Crop Science Department, North Carolina State University, Raleigh, NC 27695,; ‡ Department of Crop and Soil Sciences, University of Georgia, Tifton, GA 31794, and; § Department of Crop and Soil Sciences, University of Georgia, Griffin, GA 30224

**Keywords:** Bermudagrass morphology, Quantitative trait locus, QTL correspondence, QTL Cartographer, QTLNetwork

## Abstract

Bermudagrass (*Cynodon* (L.)) is the most important warm-season grass grown for forage or turf. It shows extensive variation in morphological characteristics and growth attributes, but the genetic basis of this variation is little understood. Detection and tagging of quantitative trait loci (QTL) affecting above-ground morphology with diagnostic DNA markers would provide a foundation for genetic and molecular breeding applications in bermudagrass. Here, we report early findings regarding genetic architecture of foliage (canopy height, HT), stolon (stolon internode length, ILEN and length of the longest stolon LLS), and leaf traits (leaf blade length, LLEN and leaf blade width, LW) in 110 F_1_ individuals derived from a cross between *Cynodon dactylon* (T89) and *C. transvaalensis* (T574). Separate and joint environment analyses were performed on trait data collected across two to five environments (locations, and/or years, or time), finding significant differences (*P* < 0.001) among the hybrid progeny for all traits. Analysis of marker-trait associations detected 74 QTL and 135 epistatic interactions. Composite interval mapping (CIM) and mixed-model CIM (MCIM) identified 32 main effect QTL (M-QTL) and 13 interacting QTL (int-QTL). Colocalization of QTL for plant morphology partially explained significant correlations among traits. M-QTL qILEN-3-2 (for ILEN; *R^2^* = 11–19%), qLLS-7-1 (for LLS; *R^2^* = 13–27%), qLEN-1-1 (for LLEN; *R^2^* = 10–11%), and qLW-3-2 (for LW; *R^2^* = 10–12%) were ‘stable’ across multiple environments, representing candidates for fine mapping and applied breeding applications. QTL correspondence between bermudagrass and divergent grass lineages suggests opportunities to accelerate progress by predictive breeding of bermudagrass.

Bermudagrass represents several resilient perennial species of the genus *Cynodon* (L.), which typically colonize tropical, subtropical, and warm temperate regions (Harlan and De Wet 1969). The United States (US) National Plant Germplasm System (NPGS) records eight extant species and ten varieties of the genus, following revised taxonomic classification by Harlan (1970). Common bermudagrass [*Cynodon dactylon* (L.) Pers.], the most widespread species of the genus, was introduced into the US in the mid-1700s, and has become the most prominent pasture grass of the southern states (Harlan 1970; Wu 2011). It exhibits a wide range of variation for plant characteristics, excellent adaptation and increased biomass in the warmer climates, traffic (wear) tolerance, aggressive growth and recuperative capacity, low input requirements, and drought and salinity tolerance (Beard 1972; Beard and Green 1994; Carrow and Duncan 1998; Hanna *et al.* 2013; Marcum 2008; Taliaferro 2003). Several triploid hybrids from interspecific crosses between *C. dactylon* (2n = 4x = 36) and *C. transvaalensis* Burtt Davy (2n = 2x = 18; South African bermudagrass) have found tremendous commercial success as lawn or sports turf (*e.g.*, football fields and golf courses) (Florkowski and He 2008; Hanna and Anderson 2008), primarily because they combine stress tolerance of the tetraploid with aesthetic properties of the diploid (Hanna *et al.* 2013; Taliaferro 2003).

Bermudagrass morphological traits are routinely used to assess phenotypic diversity (Anderson 2005; Kang *et al.* 2008; Wu *et al.* 2007), to develop core collections (Anderson 2005; Anderson *et al.* 2009; Jewell *et al.* 2012), and to characterize novel germplasm or cultivars (Lu *et al.* 2009; Taliaferro 2003). Owing to their high heritabilities (Wofford and Baltensperger 1985), morphological traits can be used as selection indices in breeding programs as proxies for correlated, yet much more complex traits (*i.e.*, biomass, abiotic stress tolerance). For example, canopy height, an evaluation of shoot vertical growth, constitutes a significant component of vegetative performance (*i.e.*, biomass, growth rate and habit) (Pittman *et al.* 2015; Sripathi *et al.* 2013; Wu *et al.* 2007) and can find use as an indirect selection index for improved pasture, biomass, and turf characteristics. Similarly, selection of improved wear (or traffic) tolerance can be achieved indirectly by selecting for morphological traits such as internode length (Wood and Law 1974), leaf length (Kowalewski *et al.* 2015), and leaf width (Kowalewski *et al.* 2015; Shearman and Beard 1975). Preliminary findings of Kowalewski *et al.* (2015) suggest that shorter and finer leaves result in increased wear tolerance in bermudagrass. Similarly, stolon growth rate and stolon length were found to be good predictors of establishment speed in *Cynodon* spp. (Magni *et al.* 2014), *Zoysia* spp. (Patton *et al.* 2007), and creeping bentgrass (Jones and Christians 2012).

Selection of improved bermudagrass cultivars is based on a number of morpho-phenological traits, growth attributes, and agronomic characteristics (including biotic and abiotic adaptations), and depends upon the intended end-use of the cultivar (for example, as pasture or turf) (Taliaferro 2003). While breeding bermudagrass for forage has primarily relied on traditional practices (*i.e.*, mass selection, recurrent selection), mostly to develop seeded varieties of *C. dactylon* var. *dactylon* (Taliaferro 2003), successful turfgrass breeding programs have utilized both seeded and vegetatively propagated interspecific crosses (between *C. dactylon* and *C. transvaalensis*) as well as natural and induced mutations (Hanna *et al.* 2013; Wu 2011). Rapid, vertical shoot growth is desirable in pasture for rapid regeneration and increased biomass production, while slow-growing, dwarf or prostrate, drought-resistant varieties are preferred in turf bermudagrass (Mutlu *et al.* 2015) to reduce mowing and irrigation requirements (Chen *et al.* 2009; Lu *et al.* 2008). A number of turf-bermudagrass genotypes, particularly used in golf greens, are spontaneous and irradiated dwarf mutants, particularly those of *C. dactylon x transvaalensis* interspecific hybrids. However, genetic and molecular architecture of dwarf mutation(s) in bermudagrass has not been elucidated (Abernathy 2014). Morphologically, short-statured plants, particularly dwarf mutants of bermudagrass and other grasses, have characteristically shorter leaves and internodes compared to their taller counterparts (Chen *et al.* 2016; Lu *et al.* 2009; Wu *et al.* 2007). Biologically, plant height is a sum of internode lengths from the base to the uppermost internode plus the length of the spike. Canopy height, on the other hand, encompasses stature-related features including vertical leaf growth, and is therefore a foliage characteristic. Accordingly, significant correlations have been reported among plant height/canopy height, internode length, and leaf length in bermudagrass (Wu *et al.* 2007) and allied lineages (Cui *et al.* 2011; Cui *et al.* 2002; Yu *et al.* 2014, Sripathi *et al.* 2013; Zeid *et al.* 2011). These traits often display continuous phenotypic variation, typical of multigenic control, and exhibit quantitative inheritance, which poses a substantial challenge in bermudagrass breeding. Nevertheless, significant genetic components and moderate to high heritability of these traits permit improvement of both turf and forage bermudagrass by breeding (Wofford and Baltensperger 1985; Wu *et al.* 2007).

Warm-season turfgrasses lag other crop species in the use of molecular and genomic approaches (Ebina *et al.* 2014; Hanna *et al.* 2013). This disconnect is particularly evident in Chloridoids, a clade with ∼1,300 species, in which very few genetic linkage maps and QTL analyses have been reported (Bushman and Warnke 2013; Cai *et al.* 2014; Crawford *et al.* 2016; Ebina *et al.* 2014; Guo *et al.* 2016). Most efforts to date have been invested in zoysiagrass (Cai *et al.* 2014; Guo *et al.* 2014; Holloway *et al.* 2018), and tef [*Eragrostis tef* (Zucc.) Trotter] (Demissie and Tatsuhito 2010; Yu *et al.* 2007; Zeid *et al.* 2011), and includes a whole genome draft sequence of tef (Cannarozzi *et al.* 2014) and zoysiagrass (Tanaka *et al.* 2016). Currently, *Cynodon* spp. lags these other chloridoids in genomic data and molecular breeding applications.

The first genetic linkage framework of bermudagrass (*C. dactylon x transvaalensis*) was based on heterozygosity within each parent (Bethel *et al.* 2006), mapping the chromosomes of each parent following a pseudo-testcross strategy (Grattapaglia and Sederoff 1994). The pseudo test-cross population was a first generation biparental population derived from two heterozygous parents, *viz*. T89 (PI 290869) and T574 (a selection from South Africa), and linkage mapping employed markers segregating in a testcross configuration. Recently, we updated this framework with additional simple sequence repeat (SSR) markers found within expressed sequence tags (ESTs) from sugarcane (EST-SSRs) (Khanal *et al.* 2017a) integrating SSRs with preexisting restriction fragment length polymorphisms (RFLPs) using OneMap (Margarido *et al.* 2007) (Khanal *et al.* 2017b). Similar parental linkage maps have been used for QTL detection in pseudo-testcross mapping populations of turfgrasses including creeping bentgrass, *Agrostis* spp. (Honig *et al.* 2014; Merewitz *et al.* 2014; Zhang *et al.* 2012); centipedegrass, *Eremochloa* spp. (Wang *et al.* 2014), and ryegrass, *Lolium* spp. (Barre *et al.* 2009; Inoue *et al.* 2004; Xiong *et al.* 2006). The updated bermudagrass map is suitable for exploratory QTL analysis since most of the parental genomes (*i.e.*, ∼80%) lie within 10 cM of a mapped marker (Khanal *et al.* 2017b), allowing adequate power for QTL detection (Lander and Botstein 1989). We also showed that tetraploid bermudagrass ‘is in a relatively advanced state of diploidization’ and there appears to have ‘substantial subgenome differentiation’, ‘strong preferential pairing’, and ‘disomic inheritance’, essentially providing a basis to use QTL mapping approaches used in the diploids. Further, we demonstrated the value of comparative genomics to navigate inadequately charted genomic landscapes of the chloridoids (Khanal *et al.* 2017b). Here, we detect and characterize bermudagrass QTL for one foliar (*i.e.*, canopy height), two stolon (*i.e.*, stolon internode length and length of the longest stolon), and two leaf traits (*i.e.*, leaf blade length and leaf blade width).

## Materials and methods

### Plant materials and trial management

The mapping population consisted of F_1_ hybrids from a cross between *C. dactylon* (T89; 4x) and *C. transvaalensis* (T574; 2x) as described (Bethel *et al.* 2006; Khanal *et al.* 2017b). For QTL analysis, clonal propagates of a total of 118 F_1_s and six standard control varieties (*i.e.*, Tifway, TifSport, Celebration, TifGrand, T-11, and TifTuf) were planted in randomized complete block designs with three replicates at Tifton, GA and Griffin, GA on 9 and June 12, 2010, respectively. Schwartz *et al.* (2018) provides detailed phenotypic observations made on the control varieties.

Bermudagrass plugs were used to establish plots (1m^2^). A granular starter (5N-10P-15K) fertilizer (Agrium U.S. Inc.; Denver, CO) was applied at a rate of 48.8 kg ha^−1^ during establishment and subsequent ammonium nitrate (34N-0P-0K) (Blue Belt Fertilizer Company; Miami, Fl) fertilizer applications were applied monthly at a rate of 24.4 kg ha^−1^ for maintenance. Plots were mowed at 3.61 cm with a rotary mower once a week throughout the project period, except for three week periods when we collected phenotypic data, and clippings were returned to the plot. Irrigation was applied immediately after each fertility application and as needed after mowing to prevent drought stress. ChipCo TopChoice Insecticide (Bayer Environmental Science; Research Triangle Park, NC) was applied once a year for the control of Fire Ants (*Solenopsis* spp.).

### Phenotypic analysis

Seventeen morpho-phenological traits were recorded over a three-year period (*i.e.*, 2010-2012) at Tifton and Griffin. A subset of morphological traits, particularly stolon traits (stolon internode length and length of the longest stolon) and foliage characteristics (canopy height, leaf blade length, and leaf blade width), were used in the current study. Canopy heights (HT) were measured (in cm) four times at Tifton (2 and July 16, 2010, July 12, 2011, and July 9, 2012) and thrice at Griffin (2 and July 16, 2010 and July 6, 2012), combining 2010 measurements within locations. Turfgrass canopy heights were measured from the ground to the top of the leaves. Stolon internode length (ILEN) was measured twice in 2011 (20 April at Tifton and 2 June at Griffin) and twice in 2012 (24 September at Tifton; 6 July at Griffin). Measurements from three subsamples of fully elongated internodal distance (in mm) of prostrate stems (between third and fourth node of the apical meristem) were averaged across the three replications of each genotype to record ILEN (*i.e.*, nine independent measurements). Length of the longest stolon (LLS; in mm) was recorded twice at Tifton (2 and July 15, 2010) and twice at Griffin (2 and July 16, 2010) during the active growing season. Leaf blade length (alternatively, leaf length or LLEN) was recorded once at both locations in 2012 (11 July at Tifton and 10 July at Griffin). Leaf blade width (alternatively, leaf width or LW) was recorded twice at Tifton (July 13, 2011 and July 11, 2012) and once at Griffin (July 10, 2012). Measurements from three subsamples of first fully expanded leaves (in mm) of a mature phytomer in the turfgrass canopy were averaged across three replicates to record LLEN and LW. First measurements of HT and LLS (*i.e.*, 2010) may not represent entire plot as they were scored around a month after planting.

Morphological data were analyzed using the GLM (general linear model) procedure in SAS 9.4 (SAS^,^ SAS Institute Inc., Cary, NC). The model consisted of the main effects of genotype, location, and/or year or time, and interactions among these main effects. Phenotypic distributions, descriptive statistics, and correlation coefficients between traits were obtained using JMP 12.1.0 (JMP, SAS Institute Inc., Cary, NC).

### Genetic linkage maps and QTL analyses

In the present study, we used EST-SSR markers, SSR-enriched genetic linkage maps, and associated genotypic data from our companion studies (Khanal *et al.* 2017a; Khanal *et al.* 2017b), which describes the nomenclature of marker names, cosegregating groups (CGs), linkage group (LGs) and homologous groups (HGs). Biparental and double-dose markers were excluded from interval mapping.

A variety of tools and techniques were explored to decipher candidate QTL underlying complex morphological traits. First, single marker analysis and digenic and trigenic interactions were pursued, the results of which were compared with those from composite interval mapping (CIM) and mixed model-based CIM (MCIM), as follows:

#### Single marker analysis (SMA):

Eighteen single environment phenotypes (*i.e.*, trait, location, and/or year, or time) pertaining to five morphological traits were independently tested for marker-trait associations against parent-specific markers (T574 or T89). Both simplex markers (*i.e.*, segregating in 1:1 ratio) including biparental markers (*i.e.*, segregating in 3:1 ratio), yielding ‘S-QTL’; and duplex markers (*i.e.*, segregating in 5:1 ratio), yielding duplex-QTL (‘D-QTL’) were used to test the contrast between genotypes (*i.e.*, presence *vs.* absence of an allele) at each marker locus using JMP (at *P* = 0.005). Detailed methodology for SMA is provided in a Supplementary Text File.

#### QTL interactions in genetic variation:

For each single environment phenotype, epistatic associations between two or three loci were extracted using the GMM algorithm implemented in software Genotype Matrix Mapping (GMM) ver. 2.1 (Isobe *et al.* 2007). We used default search parameters for testing significant associations at combinations of up to three loci and reported marker combinations represented with at least two genotypes. Three independent runs were carried out for each trait (two parent-specific runs and a combined analysis) and all categories of markers (linked or unlinked, simplex or duplex) were assessed for their contribution to the trait. Significant higher order interactions, assessed based on relative F-scores, were favored over the effects of their individual constituents.

#### Composite interval mapping:

The most likely locations of QTL and their genetic effects were detected separately in each parental linkage map by using CIM (Zeng 1994a; Zeng 1994b) employed in software WinQTL Cartographer, version 2.5 (Wang *et al.* 2007). Single environment phenotypes were used for QTL detection in HT and stolon traits, while combined (across locations and/or years) environment phenotypes were also used for leaf traits. CIM was performed using *Model 6* (Standard Model) with 5 *control markers* as cofactors (as set by *backward regression method*), a *window size* of 10 cM, and threshold value set by permutations (1,000 times) with genome-wide type I error of 5% (Doerge and Churchill 1996). Likelihood ratio (LR) test statistics was calculated at every 2 cM grid position. For closely linked peaks (*i.e.*, within 20 cM), the one with the highest LOD was reported (Tanksley 1993). If QTL (*i.e.*, 2 LOD support intervals) overlapped for a trait in different environments, they were considered coincident and reported as the same QTL (Weinig *et al.* 2002). Further, QTL detected at lower LOD thresholds (as low as two), if present, were also reported if LOD reaches the permutation-based threshold in at least one of the environments. QTL identifiers included abbreviations of the QTL ‘*q*’ followed by the trait name, its homology group, and ascending numeric values for the number of QTL in the homology group for the same trait.

#### Mixed-model based CIM:

We also performed QTL analysis with a mixed linear model (MLM) approach using MCIM provided in the software QTLNetwork ver. 2.0 (Yang and Zhu 2005; Yang *et al.* 2007). Single or combined environment phenotypes were used to detect additive effects (*α*) for main effect QTL (M-QTL) and additive by additive interactions (*αα*) for epistatic QTL (int-QTL). Further, joint analyses of multi-environment phenotypic values were independently run to decipher QTL X environment interactions (QTLx*E*). Significance of QTL detection (*i.e.*, critical F value) was determined with 1,000 permutations at genome-wide type I error of 5%. *Genome Scan Configuration* was set at 10 cM of *Testing Window Size*, 2 cM *Walk Speed*, and 10 cM *Filtration Window Size* with epistatic interactions evoked with *2D Genome Scan*.

### Comparative QTL analyses

Detected QTL were compared with reported studies in other grass species, particularly by targeting QTL identified for the traits of interest (*i.e.*, plant height, stolon internode length, and leaf length and width). Co-localization among QTL were assessed based on a comparative mapping approach employed in our companion study (Khanal *et al.* 2017b), where we juxtaposed bermudagrass markers against putative orthologous sequences (*i.e.*, sequenced RFLP probes, EST sequences) in rice and sorghum homologs. Thereafter, genomic coordinates of significant marker-trait associations were compared against reported QTL anchored to physical positions along rice and sorghum genomes, which were accessible through online databases Q-TARO (Yonemaru *et al.* 2010) and CSGRqtl (Zhang *et al.* 2013), respectively. Specifically, BLASTn based homology assessment employed in Q-TARO was used to anchor bermudagrass markers to corresponding rice QTL. Similarly, putative orthologous sequences in the sorghum genome identified using significant BLASTn hits (https://blast.ncbi.nlm.nih.gov/Blast.cgi; web accessed: 28 September 2016) were queried for anchored QTL in CSGRqtl. QTL identifiers followed respective database nomenclatures.

### Data availability

Supplemental tables and figures referenced in the manuscript are available at FigShare: https://doi.org/10.25387/g3.7834562. File S1 contains phenotype (S1.1) and genotype (S1.2) data. File S2 contains other supplemental tables and figures referenced in the results section of the manuscript.

## Results

### Field trait analysis

**Figure 1:**
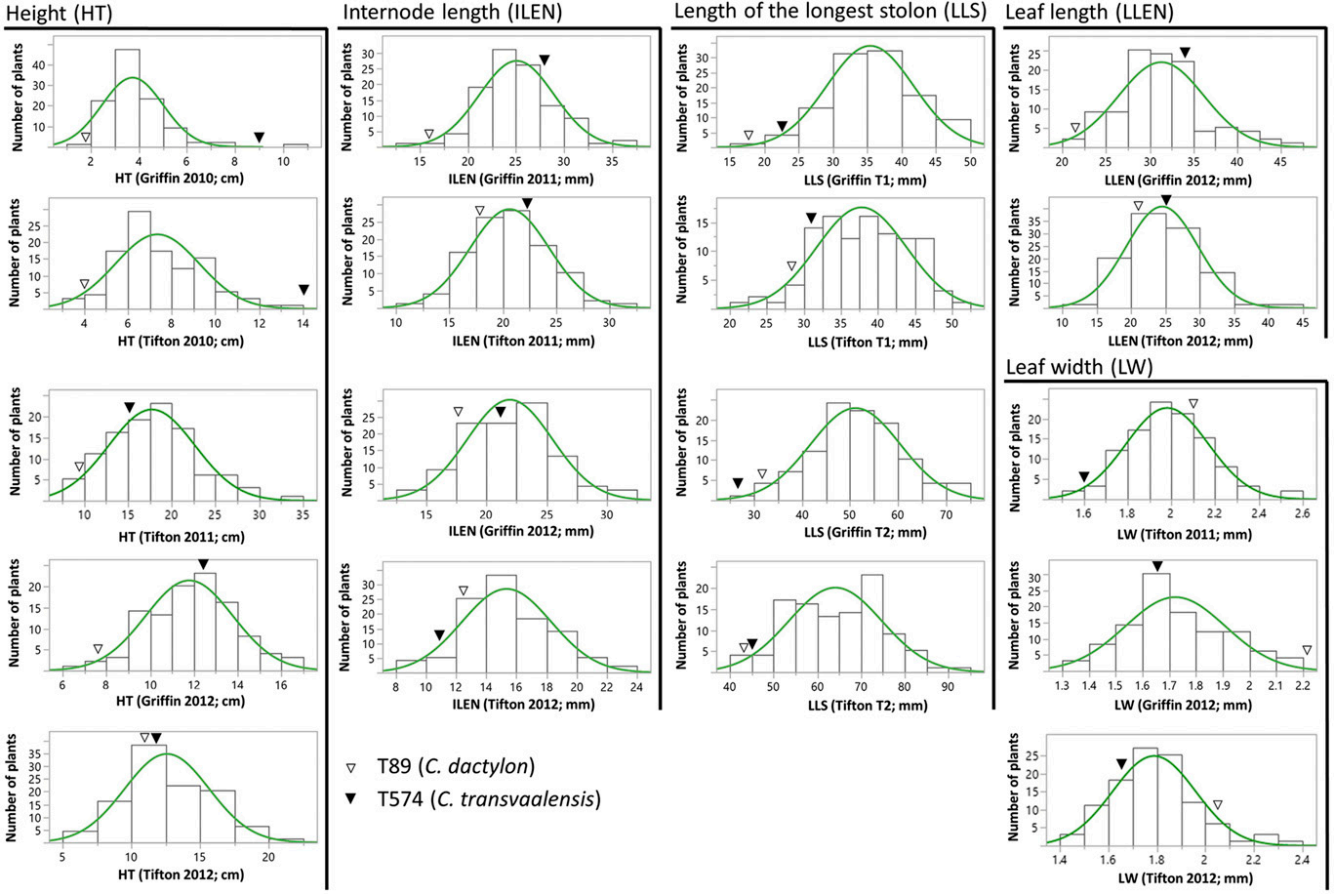
Distribution of morphological traits in clonal propagates of *C. dactylon* x *transvaalensis* intraspecific hybrids in different environments. Environments include: locations (*i.e.*, Tifton and Griffin), year (2010, 2011, and 2012), and time (T1 and T2 in 2012 for LLS). Parental means in different environments are marked by *solid* (T574) and *open* (T89) triangles.

The distributions of HT, ILEN, and LLS were approximately normal (except HT at Griffin 2010) (Shapiro and Wilk test; *P* > 0.05) and typical of quantitative (*i.e.*, polygenic) inheritance (Figure 1), while LLEN at both locations and LW at Tifton were not normal (*i.e.*, not significant at *P* < 0.01). In general, the diploid parent (T574) showed higher trait values for HT, ILEN, LLS, and LLEN, while the tetraploid parent (T89) had characteristically higher mean leaf width at both locations (Table 1). Further, trait values for LLS increased (*i.e.*, distribution shifted right), while those for ILEN decreased in the second year. Transgression was not uniform, both in terms of recurrence (*i.e.*, in different environments) and direction (*i.e.*, positive or negative). For example, while most hybrids showed higher LLS values than the parents (*i.e.*, positive transgression) in all four environments, transgression for HT was only evident in 2012. ILEN, LLEN and LW, on the other hand, had different proportions of transgressive segregants in different environments.

**Table 1:** A summary statistics of morphological traits in F_1_ mapping population and parental lines used for QTL mapping in *C. dactylon* x *transvaalensis*

Trait*^a^*	Env.*^b^*	Parental means		F_1_ population	
		T89	T574	Mean (s.d.)	Range
HT	G10	1.83	8.99	3.74 (1.3)	1.83-10.33
	T10	3.99	14.16	7.35 (1.9)	3.67-13.00
	T11	9.80	15.10	17.73 (4.9)	8.22-33.11
	G12	7.60	12.33	11.78 (2.0)	6.67-16.00
	T12	11.00	11.66	12.58 (3.1)	5.33-20.33
ILEN	G11	16.22	27.77	25.07 (3.9)	14.89-36.22
	T11	17.66	22.77	20.63 (3.7)	10.89-30.56
	G12	17.54	21.53	21.94 (3.5)	13.44-31.47
	T12	12.18	10.72	15.34 (2.9)	8.41-23.24
LLS	G10-T1	17.66	22.66	35.42 (6.3)	19.67-49.67
	T10-T1	28.00	31.66	37.80 (6.1)	22.00-50.00
	G10-T2	31.66	26.66	51.11 (9.3)	29.67-74.67
	T10-T2	43.00	45.00	64.13 (10.6)	41.00-90.33
LLEN	G12	22.74	34.35	31.31 (4.9)	20.96-45.88
	T12	21.13	25.02	24.53 (5.2)	14.19-44.07
LW	T11	2.16	1.61	1.98 (0.2)	1.54-2.59
	G12	2.26	1.64	1.72 (0.2)	1.34-2.19
	T12	2.06	1.66	1.79 (0.2)	1.44-2.39

a *HT* Canopy height (in cm, five environments), *ILEN* internode length (in mm, four environments), *LLS* length of the longest stolon (in mm, four environments), *LLEN* leaf length (in mm, two environments), and *LW* leaf width (in mm, three environments).

b Environment identified with prefixes *G* Griffin and *T* Tifton followed by *10* (2010), *11* (2011), *12* (2012) and T1 (1^st^ date) or T2 (2^nd^ date).

The effects of genotype, location, and/or year, or time and their interactions were estimated for five bermudagrass morphological traits based on the general linear models (see “Materials and methods”) in combined analysis of variance across environments. Significant differences were found among the clones (*P* < 0.001) for each of the five traits. Further, *G*x*E* interactions were significant for HT and stolon traits but not for leaf traits. Specifically, *genotype* x *location* (environment) interaction was not significant for the leaf traits *i.e.*, LLEN and LW (*P* = 0.258 and *P* = 0.852, respectively), while all relevant *G*x*E* interactions (*i.e.*, *genotype* x *location*, *genotype* x *year*/*time* etc.) were significant for HT and stolon traits. As such, QTL analyses using single environment phenotypes (*i.e.*, by location and/or year, or time) were better suited than average values (*i.e.*, across locations and/or years, or times) for HT, ILEN and LLS. On the other hand, insignificant *G*x*E* estimates for leaf traits provided an opportunity to use means across locations and/or years (*i.e.*, overall values), potentially increasing the power to detect QTL with small effects (Kong *et al.* 2014). However, we also ran joint analyses for individual traits where location and/or year, or time were included in the QTL models to provide a formal statistical basis for QTL stability instead of recurrence of coincident QTL peaks for single environment phenotypes.

### Trait correlations

Many single environment phenotypes were significantly correlated with each other (Supplemental File 2, Table S2.1). All correlations among single environment phenotypes of stolon (ILEN and LLS) and leaf characteristics (LLEN and LW) were moderately significant (*P* < 0.001). However, only seven of ten correlation coefficients among five different environments for HT were significant. Between different traits, most single environment phenotypes of stolon and leaf characteristics were significantly correlated, while a few between HT and the other traits were also significant. However, correlations among LLS and leaf characteristics were not significant. All significant correlations between traits were positive.

### Single marker analysis (SMA)

#### Marker-trait associations:

Considering single environment phenotypes, a total of 98 associations were significant at *P =* 0.005, of which 28 (28.5%) were also significant at *P =* 0.001 (Supplemental File 2, File S2.1 and Tables S2.2 and S2.3). Based on criterion set for selecting among significant markers within a CG (see “Supplemental File 2”), 19, 53, and two associations at 18 T574, 33 T89, and two biparental marker loci, respectively, were recorded as S-QTL (Supplemental File 2, Tables S2.3 and S2.4).

Possible associations between 70 duplex markers and the five traits were tested by ANOVA (as for simplex markers). Considering all the traits, 14 significant associations (at *P =* 0.005) at 11 duplex markers were detected, of which eight associated with single environment phenotypes and three with traits in two different environments (Supplemental File 2, Tables S2.2 and S2.3). Duplex markers could not be reliably mapped in the genetic linkage maps, but can be superimposed to their closest linkages. Accordingly, four of the 11 duplex markers corresponded to three different T89 CGs. One of the superimposed markers (T5746F07) coincided with an S-QTL at locus T5746H09 mapped to linkage group T89 1c/d. The remaining associations were duplex specific, with the total of 10 duplex markers adding 13 new putative genetic factors (*i.e.*, D-QTL) (Supplemental File 2, Table S2.4).

#### Consistency of S-QTL and D-QTL detection:

The consistency of QTL detection for five bermudagrass morphological traits across two (for LLEN), three (for LW), four (for ILEN and LLS), or five (for HT) environments was primarily assessed based on deduced S-QTL and D-QTL detected at a significance threshold of *P =* 0.005. The 74 deduced S-QTL were tagged at 58 marker loci, 45 of which were associated with single environment phenotypes while 10 were associated with two, and one each with three and four trait-environments, respectively. Only six S-QTL pairs tagged at five marker loci were consistent across two different environments for the same trait. Specifically, CA265902-300 was significantly associated with ILEN and LW, CA100438-215 with LLS, and CA162523-100 and CF574110-150 with HT. On the other hand, three pairs of D-QTL were associated with two different environments for the same traits (T5741E11b with HT, T5741E08 with ILEN, and T5746F07 with LLS). All nine consistent QTL (*i.e.*, six S-QTL and three D-QTL) were contributed by the T89 parent.

*A priori* knowledge of significant marker-trait association in one environment provides a rationale for employing a less stringent threshold (*i.e.*, *P =* 0.05) to identify additional marker-trait associations in different environments. Compared to the total of nine consistent QTL at *P =* 0.005, we found 11, 11, eight, six, and nine recurring QTL for HT, ILEN, LLS, LLEN, and LW, respectively, which were each detected in at least two environments with *P =* 0.005 in at least one of the environments (Supplemental File 2, Table S2.4). Further, two QTL for HT, five for ILEN, two for LLS, and four for LW were consistent across three environments each. Similarly, six LLEN QTL were detected in both environments studied (*i.e.*, Griffin and Tifton).

### Epistatic interactions

#### Interactions among digenic and trigenic int-QTL:

Two (digenic) and three (trigenic) locus combinations were evaluated for epistatic interactions on the measured traits. GMM detected a number of significant combinations (Supplemental File 2.4, Table S2.5 and Figure S2.1). For two locus combinations, 20 int-QTL were detected for HT, 12 for ILEN, 16 for LLS, 11 for LLEN, and 14 for LW. For three locus combinations, 21 int-QTL were detected for HT, 11 for ILEN, 21 for LLS, six for LLEN, and three for LW. Both parental species contributed equal numbers of int-QTL (57 T574 and 57 T89). Fifteen of the 73 two locus combinations (20%) and six of the 61 three locus combinations (∼10%) involved interactions among loci contributed by different parental species. int-QTL were distributed over 13 of 14 T574 CGs and 29 of 34 T89 CGs.

Digenic interactions explained from 16 to 61% of phenotypic variation (*i.e.*, *R*^2^) for HT, 21–78% for ILEN, 16–56% for LLS, 16–44% for LLEN, and 14–47% for LW. *R*^2^ for trigenic interactions were 43–77% for HT, 46–69% for ILEN, 33–79% for LLS, 39–80% for LLEN, and 53–71% for LW. Hence, trigenic interactions showed highest *R*^2^, except that a digenic interaction for ILEN explained more variation (78%) than any three locus combination (69%). Of the total of 135 digenic and trigenic associations, 85 (63%) contained at least one marker with a significant main effect (*i.e.*, S-QTL or D-QTL, *P* ≤ 0.05) while 50 (37%) did not involve markers with significant main effects.

#### Consistency of int-QTL detection:

Only one digenic combination involving T574 loci [*i.e.*, T5742A10b(a-) and CA093658-240(a-)] was consistently detected for HT in two different environments (*i.e.*, Griffin and Tifton in 2010). Nevertheless, GMM identified different numbers of common loci among single environment phenotypes for different traits. For HT and LLS, one locus was common across four [RZ574(a-)] and three [RZ717(a-)] different environments, respectively; however, these loci were involved in multiple digenic and trigenic associations [*i.e.*, seven for RZ574(a-), six for RZ717(a-)]. A total of four loci for HT, five for ILEN, 11 for LLS, one for LLEN, and two for LW were involved in a total of 10, 17, 27, three, and five associations, respectively, in two different environments.

### Composite interval mapping (CIM)

**Figure 2:**
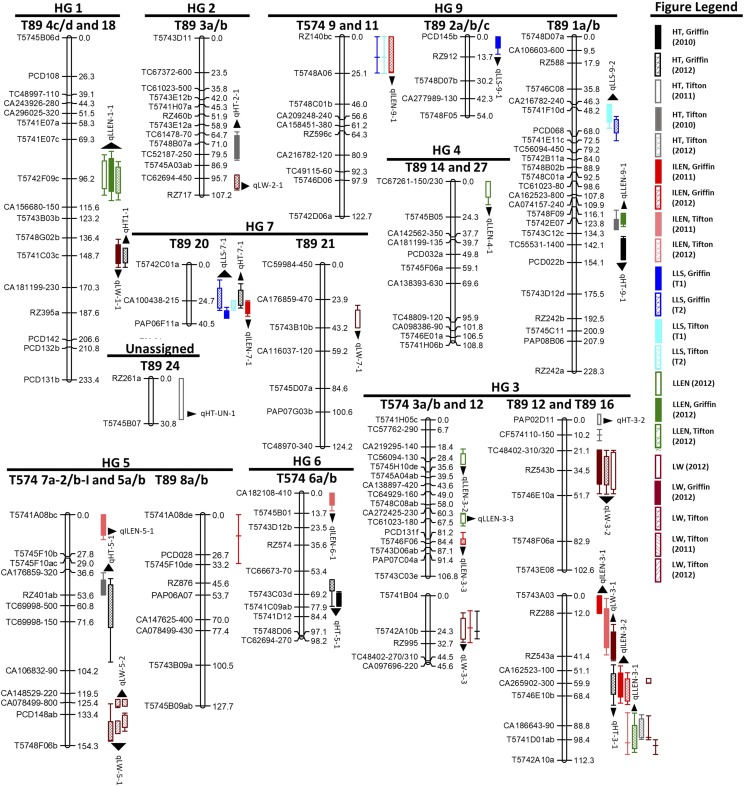
A bermudagrass genetic linkage map (*left-hand side*) superimposed with candidate genomic regions (*right-hand side*) detected for turf morphological traits. In the parental genetic linkage maps (*i.e.*, *T574* and *T89*), composite interval mapping (CIM) identified a number of significant QTL (M-QTL) for plant height (HT), internode length (ILEN), length of the longest stolon (LLS), leaf length (LLEN), and leaf width (LW). QTL are specified with LOD support intervals identified with *rectangular boxes* (1-LOD) with *whiskers* (2-LOD) and identified with *black arrowheads*. Putative homo(eo)logous regions harboring suggestive QTL (*i.e.*, LOD as low as 2) are identified using *colored* line segments corresponding to associated M-QTL and span 2-LOD intervals. Each linkage group is identified with its parental source and assigned homology group (*i.e.*, HG).

QTL Cartographer identified zero to three QTL for single environment phenotypes for each trait, with totals of seven and 21 QTL contributed by T574 and T89, respectively (Table 2, Figure 2). Using permutation based thresholds (at 1,000 permutations and genome-wide significance level of 0.05) obtained independently for each single environment phenotype, only three QTL were found to be consistent. Specifically, QTL mapped to T89 16 (qILEN-3-2), T89 20 (qLLS-7-1), and T89 12 (qLW-3-2) for ILEN, LLS, and LW, respectively, were significant in two different environments. With a modified parameter to accommodate thresholds as low as LOD = 2 for recurring trait-specific QTL (see “Materials and Methods”), we identified a total of 28 QTL (41 putative associations delineated by 31 marker intervals), 11 of which were recurrent for the five traits.

**Table 2:** Composite interval mapping of bermudagrass morphological traits in *C. dactylon* (T89) x *transvaalensis* (T574) F_1_ population

QTL	Source	Linkage group*^a^*	Env.*^b^*	Nearest marker	Position	Marker interval*^c^*	LOD	Effect	*R*^2^ (%)
**Height (HT)**									
qHT-1-1	T89	4c/d-I and 18	T10	T5741C03c	146.4	T5748G02b - CA181199-230	3.9	−1.65	15.4
qHT-2-1	T89	3a/b	G12	TC52187-250	79.0	T5743E12a - T5745A03ab	3.4	−1.42	12.6
qHT-3-1	T89	16	T10	CA265902-300	59.9	RZ543a - CA186643-90	3.0†^d^	−1.22	9.2
			T12	CA186643-90	90.8	T5746E10b - T5741D01ab	4.3	+2.49	15.2
qHT-3-2	T89	12	T11	PAP02D11	0.0	PAP02D11 - CF574110-150	3.9	−3.71	12.7
			G12	CF574110-150	10.0	PAP02D11 - TC48402-320	2.2†	+1.09	7.5
qHT-5-1	T574	7a-2/b-I and 5a/b	T10	RZ401ab	52.6	CA176859-320 - CA106832-90	3.6	+1.51	14.9
			G12	RZ401ab	50.6	CA176859-320 - TC69998-500	2.6†	+1.68	15.9
qHT-6-1	T574	6a/b	G10	T5743C03d	69.2	TC66673-70 - T5741C09ab	2.2†	−0.80	9.6
			T10	T5743C03d	61.4	TC66673-70 - T5741C09ab	3.1	−1.89	24.1
qHT-7-1	T89	20	T10	CA100438-215	24.7	T5742C01a - PAP06F11a	4.7	−1.53	15.6
qHT-9-1	T89	1a/b	G10	TC55531-1400	142.2	T5743C12c - PCD022b	2.3†	−0.72	7.5
			G12	T5742E07	127.8	T5748F09 - T5743C12c	3.6	+1.86	21.4
qHT-UN-1	T89	24	T11	T5745B07	18.1	RZ261a - T5745B07	3.6	+4.54	18.1
**Internode length (ILEN)**									
qILEN-3-1	T89	16	G11	T5743A03	0.0	T5743A03 - RZ543a	2.4†	+2.40	9.4
			G12	RZ288	24.1	T5743A03 - RZ543a	3.5	+3.16	19.5
qILEN-3-2	T89	16	G11	CA265902-300	59.1	RZ543a - CA186643-90	4.0	−2.64	11.3
			T11	CA265902-300	63.9	CA162523-100 - CA186643-90	5.1	−3.33	19.6
qILEN-3-3	T574	3a/b and 12	T11	T5746F06	84.4	TC61023-180 - T5743D06b	6.2	−3.52	21.6
qILEN-5-1	T574	7a-2/b-I and 5a/b	G12	T5741A08bc	8.0	T5741A08bc - T5745F10b	3.6	+3.46	23.3
qILEN-6-1	T574	6a/b	G12	CA182108-410	0.0	CA182108-410 - T5745B01	3.8	−2.62	13.2
qILEN-7-1	T89	20	G11	CA100438-215	30.7	T5742C01a - PAP06F11a	3.4	−3.70	22.4
qILEN-9-1	T574	9 and 11	T11	RZ140b	12.0	RZ140bc - T5748A06	3.8	−3.22	18.5
**Length of the longest stolon (LLS)**									
qLLS-7-1	T89	20	GT1	CA100438-215	34.7	CA100438-215 - PAP06F11a	3.3†	−6.07	22.9
			TT1	CA100438-215	24.7	T5742C01a - PAP06F11a	4.5	−4.49	13.4
			TT2	CA100438-215	26.7	T5742C01a - PAP06F11a	7.1	−11.24	27.2
qLLS-9-1	T89	2a/b/c	GT1	PCD145b	0.0	PCD145b - RZ912	4.0	−5.21	14.2
qLLS-9-2	T89	1a/b	TT1	PCD068	60.2	T5741F10d - T5741E11c	4.5	+6.05	24.4
			GT2	T5741F10d	48.2	T5746C08 - PCD068	2.6†	+5.85	9.1
**Leaf length (LLEN)**									
qLLEN-1-1	T89	4c/d-I and 18	G/T12	T5742F09c	96.2	T5741E07c - CA156680-150	3.8	−3.10	11.5
			G12	T5742F09c	93.3	T5741E07c - CA156680-150	2.7†	−3.25	10.6
			T12	T5742F09c	96.2	T5741E07c - CA156680-150	4.0	−3.75	11.7
qLLEN-3-1	T89	16	T12	T5741D01ab	96.8	T5746E10b - T5742A10a	3.4	+3.35	9.8
qLLEN-3-2	T574	3a/b and 12	G/T12	TC56094-130	28.4	CA219295-140 - T5745H10de	4.0	−3.72	14.7
qLLEN-3-3	T574	3a/b and 12	G/T12	TC61023-180	66.3	CA272425-230 - PCD131f	3.9	−4.58	23.8
qLLEN-4-1	T89	T89 14 and 27	G/T12	TC67261-150/230	0.0	TC67261-150/230 - T5745B05	3.5	−2.93	10.6
qLLEN-9-1	T89	1a/b	G12	T5742E07	122.2	T5748F09 - T5743C12c	3.9	+3.89	14.8
**Leaf width (LW)**									
qLW-1-1	T89	4c/d-I and 18	G12	T5741C03c	148.4	T5748G02b - CA181199-230	3.6	+0.15	14.9
qLW-2-1	T89	3a/b	T12	TC62694-450	95.8	T5745A03ab - RZ717	3.4	−0.11	10.0
qLW-3-1	T89	16	G/T12	CA265902-300	59.1	CA162523-100 - CA265902-300	4.1	−0.11	13.6
			G12	RZ543a	36.1	RZ288 - CA162523-100	4.5	+0.18	21.3
qLW-3-2	T89	12	G/T12	RZ543b	31.1	CF574110-150 - T5746E10a	3.4	−0.10	11.9
			G12	TC48402-320	27.1	CF574110-150 - RZ543b	3.6	−0.13	12.0
			T12	RZ543b	34.5	CF574110-150 - T5746E10a	3.4	−0.11	10.3
qLW-3-3	T574	3a/b	G/T12	T5742A10b	26.3	T5741B04 - RZ995	3.3	+0.14	23.3
qLW-5-1	T574	7a-2/b-I and 5a/b	T11/12	PCD148ab	141.4	PCD148ab - T5748F06b	3.4	−0.18	33.5
			T12	PCD148ab	143.4	CA078499-430/600 - T5748F06b	2.2†	−0.16	20.9
			T11	PCD148ab	135.4	PCD148ab - T5748F06b	2.8†	−0.14	13.3
qLW-5-2	T574	7a-2/b-I and 5a/b	T11/12	CA078499-430/800	125.5	CA148529-220-PCD148ab	4.1	−0.12	13.9
			T11	CA078499-430/800	125.5	CA148529-220-PCD148ab	5.3	−0.17	16.8
qLW-7-1	T89	21	G/T12	T5743B10b	37.9	CA176859-470 - T5743B10b	4.3	−0.14	22.7

a Linkage group nomenclature following our companion paper. ^b^ Environment identified with prefixes *G* Griffin and *T* Tifton followed by *10* (2010), *11* (2011), *12* (2012) and T1 (1^st^ date) or T2 (2^nd^ date). ^c^ Markers spanning 2-LOD support interval. ^d^ QTL that do not meet permutation threshold and are nominally significant (LOD = 2).

#### Height (HT):

For HT data recorded in five separate environments (*i.e.*, three years at Tifton and two years at Griffin), a total of nine putative QTL (two T574 and seven T89) were identified, explaining 7.5–24% of the phenotypic variation (avg. 14.3%). Putative recurrent QTL for HT included two mapped to T574 and three to T89 (specifically, T574 6a/b and T574 7a-2/b-I, and T89 16, T89 12, and T89 1a/b), all detected in two different environments. Notably, a number of significant and suggestive QTL peaks in homology group 3 (*i.e.*, HG 3) corresponded to syntenic regions in T574 homologs (T574 3a/b and 12) and T89 homo(eo)logs (T89 3c/d and 16 and T89 12), indicating that the region carries major QTL(s) for HT.

#### Stolon internode length (ILEN):

Four separate environments for ILEN produced a total of seven putative QTL (four T574 and three T89), which explained 9–23% of phenotypic variation (avg. 17.6%). Recurrent QTL for ILEN included two coincident QTL peaks that were mapped to T89 16 (HG 3) and detected in two different environments. In fact, T89 16 carried QTL at almost overlapping 2 LOD support intervals (*i.e.*, confidence interval) in all four environments, indicating that the linkage group harbors major ILEN QTL. This is further supported by the fact that a suggestive QTL was also detected in T574 at a homologous region (T574 3a/b and 12). The homologous region also corresponds to one explaining significant phenotypic variation for HT. Further, a significant QTL peak in T574 7a-2/b-I and 5a/b and a suggestive peak in T89 8a/b could be associated with the same locus in the two genomes.

#### Length of longest stolon (LLS):

For LLS data collected twice at Griffin and Tifton during 2012, a total of three putative QTL were detected, all three of which were contributed by T89. These M-QTL explained 9–27% of phenotypic variance. Further, three coincident QTL peaks in T89 20 were detected in three different environments explaining 13–27% of total phenotypic variation for single environment phenotypes, suggesting that it is a site for a major QTL underlying LLS in bermudagrass. Further, a number of suggestive QTL peaks were contributed by T89 homo(eo)logs constituting HG 6. Although significant QTL were not contributed by T574, three suggestive QTL were recurrent in two environments each. Interestingly, these QTL seemingly have suggestive T89 counterparts at homologous regions, indicating that a number of small effect QTL (R2 < 10%) and a large effect one (T89 20) influence LLS in bermudagrass.

#### Leaf length (LLEN) and leaf width (LW):

Leaf trait characteristics recorded at Griffin (2012) and Tifton (2011 and 2012) showed a total of 14 putative QTL, five from T574 and the rest from T89. For LLEN, four QTL from T89 explained 10–15% of phenotypic variation and a pair of coincident peaks (constituting QTL qLLEN-1-1) was recurrent in both locations (Griffin and Tifton) as well as in the across-location estimated mean. A total of eight putative QTL (three T574, five T89) for LW explained 10–33.5% of total phenotypic variation. A pair of coincident QTL peaks was mapped to T89 12 and was recurrent in both locations as well as in the across-location means. Multiple overlapping QTL peaks (mapped to T574 7a-2/b-I and 5a/b) represented a T574 QTL for LW, the most prominent one of which explained 33.5% of variation for the trait.

### Mixed-model based CIM

#### Additive effects and additive x E interactions:

Separate analysis of single environment phenotypes detected a total of seven unique M-QTL, four for ILEN, one for LLS, and two for LW (Table 3). No QTL were detected for HT and LLEN based on input parameters for QTL detection (see “Materials and Methods”). Five of the seven M-QTL were contributed by T89, and two by T574. Five of the seven QTL were also detected by CIM and were identified with CIM QTL identifiers, while two novel QTL specific to ICIM were assigned new identifiers (*i.e.*, qILEN-1-1 and qILEN-3-4). Most QTL alleles conditioned an increase in trait values (suggesting that the dominant alleles decreased trait values) except that qILEN-1-1 decreased internode length.

**Table 3:** Additive (*α*) and additive x environmental (*αe*) effects underlying bermudagrass morphology detected by mixed-model based CIM (MCIM) procedure implemented in QTL Network v. 2.0

Trait	QTL	Env.*^a^*	Source	Interval	Linkage group*^b^*	*α**^c^*	*αe1**^d^*	*αe2^e^*	*αe3^f^*	*αe4*^g^	*R*^2^*_α_* (%)*^h^*	*R*^2^*_αe_* (%)^i^
ILEN	qILEN-3-2	Joint	T89	CA265902-300 - T5746E10b	16	−2.02****	NS	NS	NS	+0.79*	5.1	0.7
	qILEN-1-1†*^j^*	G11	T89	T5743D12e - PCD021b	4c/d-1 and 18	+2.63****					25.6	
	qILEN-3-2	G11	T89	CA265902-300 - T5746E10b	16	−3.10****					15.6	
	qILEN-3-2	T11	T89	CA265902-300 - T5746E10b	16	−3.65****					17.6	
	qILEN-3-3	T11	T574	T5746F06 - T5743D06b	3a/b and 12	−3.56****					17.5	
	qILEN-3-4†	T12	T89	T5741D01a - T5742A10a	16	−2.03****					11.1	
LLS	qLLS-7-1	Joint	T89	CA100438-215 - PAP06F11a	20	−1.91**	+2.66**	NS	NS	−4.35****	3.2	1.2
	qLLS-7-1	TT1	T89	CA100438-215 - PAP06F11a	20	−5.11****					14.8	
	qLLS-7-1	TT2	T89	CA100438-215 - PAP06F11a	20	−10.73****					23.1	
LW	qLW-2-1	T12	T89	TC62694-450 - RZ717	3a/b	−0.13****					12.7	
	qLW-5-1	T12	T574	CA078499-800 - PCD148a	7a-2/b-I and 5a/b	−0.10****					10.7	

a Single environment phenotypes identified with prefixes *G* Griffin and *T* Tifton followed by *10* (2010), *11* (2011), *12* (2012) and T1 (1^st^ date) or T2 (2^n^d date).

Joint refers to joint environment analysis.

b Linkage group nomenclature following our companion paper.

c Additive (and/or dominance) effect of a QTL.

d *αe* at Griffin (2011) for ILEN or Griffin (1^st^ date) for LLS;

e *αe* at Tifton (2011) for ILEN or Tifton (1^st^ date) for LLS;

f *αe* at Griffin (2012) for ILEN or Griffin (2^nd^ date) for LLS;

g *αe* at Tifton (2012) for ILEN or Tifton (2^nd^ date) for LLS.

h percent variation explained by ‘*α*’ effect; ^i^ percent variation explained by ‘*αe*’ effect.

j Novel QTL identified by MCIM (not detected/significant at permutation threshold following CIM).

* 0.05, **0.01, ****0.001, *NS* not significant.

Joint analysis of each trait to decipher genotype x environment (*G*x*E*) interactions detected two additive x environment effects (*αe*, Table 3). Specifically, qILEN-3-2 showed significant *αe* effect for ILEN data collected at Tifton in 2012 (*αe*4), while qLLS-7-1 showed significant interactions with LLS data collected at Griffin in 2011 (*αe*1) and Tifton in 2012 (*αe*4). The two QTL were consistently detected across two to three environments using SMA, CIM, and MCIM following single environment QTL analysis.

#### Epistatic effects and epistasis x E interactions:

Three additive x additive QTL (*αα*, Table 4) were detected for single environment phenotypes, two of which involved T89 loci. Interestingly, two of the three epistatic pairs carried M-QTL identified by CIM. Specifically, qILEN-6-1/qILEN-5-1 and qLW-3-1/qLW-3-2 showed significant *αα* interactions in G12 (*i.e.*, Griffin 2012) and all four were also detected by CIM as bearing M-QTL effect. A third *αα* interaction (*i.e.*, qLLS-5-1/qLLS-2-1) also carried a suggestive QTL qLLS-5-1 (*i.e.*, LOD = 3). As such, all three *aa* interactions involved putative M-QTL.

**Table 4:** Epistatic effects underlying bermudagrass morphology detected by mixed-model based CIM (MCIM) procedure implemented in QTL Network v. 2.0

Trait	Env.*^a^*	Source	Loci_(i)			Loci_(j)			*αα**^d^*	*ααe*1*^e^*	*ααe*2*^f^*	*ααe*3*^g^*	*ααe*4*^h^*	*ααe*5*^i^*	*R*^2^_αα_ (%)*^j^*	*R*^2^_ααe_ (%)*^k^*
			QTL_i*^b^*	Interval_i	Linkage group*^c^*	QTL_j	Interval_j	Linkage group								
HT	Joint	T574	qHT-3-3†	CA138897-420 - TC64929-160	3a/b and 12	qHT-4-1†	CA097171-420 - CA097171-280	4a/b	+1.34****	−1.09*	NS	+3.16****	NS	NS	1.1	1.9
	T11	T574	qHT-3-3†	CA138897-420 - TC64929-160	3a/b and 12	qHT-4-1†	CA097171-420 - CA097171-280	4a/b	+4.63****						16.6	
ILEN	Joint	T89	qILEN-6-2†	TC62910-300 - RZ142b	1c/d	qILEN-1-1†	PCD021b - TC48763-210	4c/d-1 and 18	−2.01****	NS	NS	NS	NS		3.4	1.2
		T574	qILEN-6-1	T5745B01 - T5743D12b	6a/b	qILEN-5-1	T5741A08bc - T5745F10b	7a-2/b-I and 5a/b	+2.02****	NS	NS	NS	NS		10.5	1.8
			qILEN-6.3†	T5741C09a - T5741D12	6a/b	qILEN-3-3	T5746F06 - T5743D06b	3a/b and 12	+2.59****	NS	+1.64*	NS	NS			
			qLEN-9-2†	RZ596c - CA216782-120	9 and 11	qILEN-4-1†	PCD032c - T5741F10a	4a/b	+1.99****	NS	NS	NS	NS			
	G12	T574	qILEN-6-1	T5745B01 - T5743D12b	6a/b	qILEN-5-1	T5741A08bc - T5745F10b	7a-2/b-I and 5a/b	+3.64****						18.6	
LLS	Joint	T89	qLLS-6-1†	TC61478-580 - TC61478-600	1c/d	qLLS-4-1†	PCD137c - TC51780-120	6a/b	+7.24****	NS	NS	NS	NS		5.3	1.7
			qLLS-9-3†	T5745D12c - T5748G06ab	2a/b/c	qLLS-7-1	CA100438-215 - PAP06F11a	20	+4.28****	NS	NS	NS	NS			
			qLLS-9-2	RZ588 - T5746C08	1a/b	qLLS-3-1†	RZ538 - TC55445-110	3c/d and 16	+5.05****	NS	NS	NS	NS			
	TT1	T89	qLLS-5-1†	T5742F07 - PAP10A04b	15	qLLS-2-1†	CA097696-140 - T5741F06c	4a/b	−7.13****						17.6	
LLEN	Joint	T89	qLLEN-6-1†	RZ900 - CA294786-340	7a/b and 11	qLLEN-4-1†	TC67261-150/230 - T5745B05	14 and 27	+4.71****	NS	NS				7.2	1.5
		T574	qLLEN-3-2†	TC56094-130 - T5745H10de	3a/b and 12	qLLEN-8-1†	CA094069-210 - T5743B03c	8 and 10	+4.54****	NS	NS				8.6	0.1
LW	Joint	T89	qLW-3-1	CA162523-100 - CA265902-300	16	qLW-3-2	TC48402-320 - RZ543b	12	+0.20****	NS	NS	NS			10.9	0.5
	T11	T574	qLW-5-1	CA078499-800 - PCD148ab	7a-2/b-I and 5a/b	qLW-3-4†	T5743D06a - PAP07C04a	3a/b and 12	+0.27****						18.7	
	G12	T89	qLW-3-1	CA162523-100 - CA265902-300	16	qLW-3-2	TC48402-320 - RZ543b	12	+0.26****						22.5	

a Single environment phenotypes identified with prefixes *G* Griffin and *T* Tifton followed by *10* (2010), *11* (2011), *12* (2012) and T1 (1^st^ date) or T2 (2^nd^ date).

Joint refers to joint environment analysis.

b QTL nomenclature following CIM; novel QTL are identified with suffix ‘†’.

c Linkage group nomenclature following our companion paper.

d Additive (and/or dominance) by additive (and/or dominance) (*i.e.*, *αα*) effect of a QTL.

e ^fghi^*ααe* for HT (5 environments), ILEN (4 environments), LLS (4 environments), LLEN (2 environments), and LW (3 environments).

j percent variation explained by ‘*αα*’ effect;

k percent variation explained by ‘*ααe*’ effect.

* 0.05, ****0.001, *NS* not significant.

In the joint analysis, a total of 22 loci were involved in epistatic interaction in 11 digenic combinations, five of which had at least one M-QTL (Table 4). For all new loci involved in epistatic interactions, new QTL identifiers were provided. Concerning estimated effects of the digenic interactions, one pair had negative and eleven pairs had positive effects, while two of the 11 interactions also displayed *αα* x *ααe* effects.

### Genomic hubs carrying colocalized QTL

In SMA analysis, eight markers mapping to six different LGs and three unlinked markers were involved in 29 associations with at least two different traits (Supplemental File 2, Table S2.4). One of the eight markers (RZ140bc) originated from T574 while the rest affiliated with T89. Interestingly, putative S-QTL effects were in the same direction in all cases. For example, three shared markers (CA162523-100, RZ900, and T5741A10a) conditioned a decrease, while one (PCD053c) increased trait values for HT and LLEN simultaneously. Further, one common digenic interaction involving T574 loci [T5745B06a(–) and T5741H06a(a-)] was detected for HT and ILEN in Tifton 2012, which conditioned a decrease in both trait values following GMM analysis. Similarly, two major genomic hubs carried multiple QTL for bermudagrass morphological traits based on CIM. HG 3 and associated LGs from both parental genomes (T574 3a/b and 12, T89 3c/d and 16, and T89 12) carried several QTL peaks associated with HT, ILEN, LLEN and LW. Similarly, T89 20 (constituting HG 7) carried recurrent QTL peaks for LLS (detected in three different environments) and two other significant QTL, one each for HT and ILEN. Significant positive correlations among five morphological traits were partially explained by the presence of colocalized QTL showing effects in the same direction. However, the genetic basis of relationships between the traits and high coincidence among QTL underlying bermudagrass morphology may be due to either pleiotropy or tight linkages and needs further assessment.

### QTL correspondence

Homology based search for anchored QTL in rice and sorghum genomes showed that at least 33 significant marker-trait associations (QTL) (corresponding to 29 markers) identified in our study colocalize with target QTL in other grasses at putative orthologous and/or syntenic regions (Supplemental File 2, Table S2.2). For example, a number of significant marker-trait associations were detected for HT at putative syntenic regions in bermudagrass LGs constituting HG 03 [*i.e.*, T574 3a/b and 12 and T89 3cd and 16], which represented one of the two genomic hubs carrying colocalized QTL. Specifically, a cross-transferable *Pennisetum* marker (*i.e.*, PAP02D11) mapped to T89 12 was significantly associated with HT at Tifton (2011). The orthologs of PAP02D11 were present in syntenic chromosomal regions of rice (*Os 1*) and sorghum (*Sb 3*) and anchored a number of plant height QTL in both, including a semi-dwarfing gene (*i.e.*, *sd-1*) in rice. Plant height QTL in sorghum (*i.e.*, Q.Lin1995.HtAvg-1-1) and sugarcane (*i.e.*, Q.Ming2002a.PLHT.11_3-1) were also associated with a cross-transferable sugarcane marker (*i.e.*, CF574110) that mapped adjacent (∼10 cM) to PAP02D11 and showed significant associations with canopy height in two different environments. Another *Pennisetum* marker (*i.e.*, PAP07C04a) which mapped to the homo(eo)logous T574 LG (*i.e.*, T574 3a/b and 12) was also anchored to the two plant height QTL tagged at CF574110 loci and showed significant association with canopy height in one of the environments (*i.e.*, Tifton 2010). Similarly, a second genomic hub with colocalized QTL (*i.e.*, HG 07) carried markers orthologous to a putative syntenic region of a sorghum chromosome (*i.e.*, *Sb 9*) anchoring sorghum plant height QTL (*i.e.*, Q.Lin1995.HtM-9-1 and Q.Brown2006.PTHT-9-1).

## Discussion

In the present study, we enhance molecular and genomic information for one of the most important warm-season turf genera, *Cynodon*, reporting QTL analyses using SSR-enriched genetic linkage maps and morphological trait data collected across two different locations and/or two to three different years, or times (*i.e.*, two to five environments). To date, only QTL analysis using biparental segregants was reported for establishment rate in common bermudagrass population (Guo *et al.* 2016). Based on an F_1_ population resulting from interspecific hybridization between common bermudagrass and African bermudagrass that have contrasting phenotypic and agronomic characteristics and condition heterotic attributes to their progenies, QTL identified in this study are directly relevant to major bermudagrass turf cultivars and are candidates for improvement in similar pedigrees.

A genetic map with DNA markers within 10 cM of most of the genome (∼80% coverage) allowed us to use SMA and GMM together with standard interval mapping approaches (*i.e.*, CIM and MCIM) to dissect complex morphological traits. Following SMA, we detected a total of 112 significant marker-trait associations (at *P* = 0.005 with estimated 8–20% false positives), 33 of which showed correspondence with anchored QTL in orthologous/syntenic regions of allied genomes. Based on corresponding associations in different environments at a lower threshold (*P* = 0.05) we deduced a total of 45 recurrent associations for the five traits, a subset of which were also detected by CIM and MCIM procedures and have promise for applied breeding applications. While most QTL conditioned a decrease in trait values, a third (*i.e.*, 27/81 QTL detected by SIM and 14/40 QTL detected by CIM or ICIM) were associated with increases, suggesting that there is scope for divergent selection in bermudagrass. The current study was based on QTL analyses in separate parental linkage maps. T89 contributed more QTL, consistent with its tetraploidy and more markers mapped to it, but each parent contributed to QTL effects in both directions for each trait under consideration. However, we could not make unequivocal assertions about QTL correspondence between T574 and T89, particularly because of a paucity of allelic homologs (*i.e.*, common markers) between the two maps, which curtailed our ability to evaluate gene dosage effects. As such, there is a need for high density integrated linkage maps built with codominant markers and biparental bridges (*i.e.*, markers segregating in 3:1 ratio), as elaborated in our companion paper (Khanal *et al.* 2017b).

### Genetic basis of bermudagrass morphological traits

Canopy height in turfgrasses is a function of both stem and leaf extension, a fact that was evident from several significant correlations between HT, stolon (particularly ILEN), and leaf traits (particularly LLEN), further supported by a number of coincident QTL among traits (more so with ILEN and LLEN). Significant positive correlation between plant height and leaf length was also reported in rice (Cui *et al.* 2002; Yan *et al.* 1999), switchgrass (Sripathi *et al.* 2013), and perennial ryegrass (Yamada *et al.* 2004), and co-localized QTL were also evident in some studies (Cai *et al.* 2015; Yan *et al.* 1999), suggesting that the genetic basis of plant height and leaf length are partially overlapping. In the current study, four markers were significantly linked with both traits - two of which were also detected by CIM. Similarly, five markers were significantly associated with HT and ILEN, while CIM detected two coincident QTL at the two genomic hubs – the result is consistent with prior studies reporting significant positive correlations partially explained by the presence of colocalized QTL [for example: tef (Yu *et al.* 2007; Zeid *et al.* 2011); wheat (Cui *et al.* 2011; Yu *et al.* 2014)]. For HT, SMA produced 17 deduced QTL (including three D-QTL; eight recurrent) and CIM/ICIM detected 11 QTL (average *R*^2^ = 13.6%) including five recurrent (*i.e.*, in at least two different environments), one int-QTL (*R*^2^*_αα_*=1.1% in joint analysis), and a significant ‘*Epistasis*x*E*’ interaction (*R*^2^*_ααe_* = 1.9%), which partially explains weak correlations, significant *GxE*, and indicates discrete and multigenic regulation of the trait in different environments - an observation reiterated in other grasses (Pauly *et al.* 2012; Poncet *et al.* 2000).

Significant correlations among single environment phenotypes of stolon characteristics indicated that we may be able to find recurrent QTL in different environments. Accordingly, a total of 24 (with 11 recurrent; three D-QTL) and eight (all recurrent including 1 D-QTL) deduced QTL were identified for ILEN and LLS. CIM/ICIM detected 13 QTL (average *R*^2^ = 13.4%) for ILEN that included two recurrent and four int-QTL (*R*^2^*_αα_*=14% in joint analysis), while seven QTL (average *R*^2^ = 13.5%) were detected for LLS that included two recurrent and four int-QTL (average *R*^2^*_αα_*=5.3% in joint analysis). Based on QTL recurrence, stolon traits were seemingly more ‘stable’ than canopy height.

Wofford and Baltensperger (1985) reported moderately high heritabilities associated with LLEN and LW in common bermudagrass. In the current study, *GxE* (*i.e.*, location) interaction for leaf traits was not significant, which is consistent with Wofford and Baltensperger (1985) and Kowalewski *et al.* (2015), and provided a statistical basis for combined data analysis across years for the two traits. We thus used separate environment phenotypes as well as combined analysis for QTL detection. Phenotypic distributions of the leaf traits (specifically, LLEN at both locations and LW at one) approached but were not exactly Gaussian (*i.e.*, normality rejected by Shapiro-Wilk test), suggesting an oligogenic mode of inheritance. However, leaf length is often characterized as a trait governed by many genes with small effects and profoundly influenced by growth environment (Barre *et al.* 2009; Barre *et al.* 2015; Marousky *et al.* 1992). In the current study, a total of 13 deduced QTL from SMA (including six recurrent QTL) and eight CIM/ICIM-detected QTL (average *R*^2^ = 12.3%) including one recurrent (repeatedly detected in separate and joint environment CIM analyses) and two int-QTL (*R*^2^*_αα_*=3% in joint analysis with MCIM) were identified for LLEN.

For LW, seventeen SMA-based QTL (including seven recurrent; four D-QTL) and nine CIM/ICIM-detected QTL (average *R*^2^ = 16.5%) including two recurrent QTL and two int-QTL (average *R*^2^*_αα_*=20.6%) were identified in the current study. In a comparable study in *Agrostis* (Zhang *et al.* 2012), three leaf width QTL collectively explained 23% of phenotypic variation. QTL effects were higher in our study, with multiple regression estimates involving two to four SMA-derived QTL explaining 27.2–69.4%, while two to three CIM-detected QTL collectively explaining 30.1–48.2%. of phenotypic variation in three different environments. A genomic region in LG 7a-2/b-I carried tandem QTL (*i.e.*, qLW-5-1 and qLW-5-2) contributed by T574 (the parent with finer leaves) that conditioned a decrease in leaf width and explained 13.3–33.5% of phenotypic variance in Tifton in separate and joint environment analyses (*i.e.*, across years). In rice, Liu *et al.* (2015) reported a presence of closely linked leaf width QTL in chromosome 3 that were stable across 5 different environments. We note that our tandem LW QTL do not correspond with the ones reported by Liu *et al.* (2015), but detection of closely linked QTL contributing significantly to the same trait in diverse lineages is noteworthy.

While a majority of QTL alleles conditioned a decrease in LLEN and LW (*i.e.*, heterozygotes with decreased trait values), regardless of their parental origin, a few also increased trait values. The hybrid bermudagrass population showed significant positive correlations for the leaf traits, despite contrasting parental characteristics, and yet common putative QTL were not detected. Significant correlations between these leaf traits have also been reported in other grass lineages (Wang *et al.* 2014; Yamada *et al.* 2004). However, QTL colocalization between leaf traits is often lacking or limited, as observed in the current study. QTL correspondence was completely lacking in perennial ryegrass (Yamada *et al.* 2004), centipedegrass (Wang *et al.* 2014), sorghum (Lu *et al.* 2011), and Brachiaria (Thaikua *et al.* 2016), while few colocalized QTL were reported in rice (Cai *et al.* 2015; Liu *et al.* 2015; Wang *et al.* 2012; Yan *et al.* 1999; Zhang *et al.* 2015a) and wheat (Wu *et al.* 2016). In a maize nested association mapping (NAM) population, leaf length and width displayed weak correlations and largely discrete putative causative variants underlying the traits (Tian *et al.* 2011). Apparently, leaf length and width share few pleiotropic or closely linked genetic determinants.

### QTL correspondence among grasses

Although many turf-bermudagrasses (particularly those used in the golf greens) are dwarf mutants, genetic architecture of dwarf mutation(s) in bermudagrass has not been elucidated (Abernathy 2014); unlike several grass lineages in which molecular and physiological basis of dwarfing are well characterized. For example, rice chromosome 1 (*Os* 1) carries a number of plant height QTL (Thomson *et al.* 2003) apparently coincident with dwarfing genes *d-10* (Huang *et al.* 1996; Yu 1991) and *d-18* (Huang *et al.* 1996; Ideta *et al.* 1993; Xiao *et al.* 1992) and a semi-dwarfing gene *sd-1* (Cho *et al.* 1994; Huang *et al.* 1996; Li *et al.* 2003). The ‘Green Revolution gene’, *sd-1* encodes a mutant enzyme involved in gibberellin synthesis and imparts short-stature (*i.e.*, semi-dwarfing) to rice (Monna *et al.* 2002; Spielmeyer *et al.* 2002). A QTL (*i.e.*, *ph1*) close to the *sd-1* locus, has been repeatedly detected in different QTL studies and two genes of interest (*i.e.*, *OsGA3ox2* gibberellin biosynthetic gene and *D2* brassinosteroid biosynthetic gene) have been identified and isolated from the vicinity of *ph1* (You *et al.* 2006). Putative orthologous regions around *sd-1* have also been reported to carry plant height QTL in maize (Austin and Lee 1996; Khairallah *et al.* 1998; Schön *et al.* 1994), *Setaria* (Mauro-Herrera and Doust 2016), sorghum (Zhang *et al.* 2015b), and switchgrass (Mauro-Herrera and Doust 2016; Serba *et al.* 2015). Notably, bermudagrass HG 3 also seems to carry gene(s) conditioning plant morphology, particularly relevant to plant stature, at regions homologous to the semi-dwarfing *sd-1* locus. Accordingly, a number of non-random correspondences of QTL locations between bermudagrass and allied grasses were detected. For example, being detected twice at Tifton (TT1 and TT2) and attaining marginal significance (LOD = 3.3) at GT1 following CIM and MCIM, qLLS-7-1 was one of the ‘stable’ QTL that conditioned a decrease in LLS and explained significant proportions of phenotypic variation (*i.e.*, 13.7–23.1%) of the trait in different environments. A canopy height QTL (*i.e.*, qHT-7-1) was also coincident with qLLS-7-1. Markers flanking the QTL region (*i.e.*, CA100438 and PAP06F11a) had putative orthologs at the syntenic region of a homologous sorghum chromosome (*i.e.*, *Sb 9*) that anchored two plant height QTL (*i.e.*, Q.Lin1995.HtM-9-1, Q.Brown2006.PTHT-9-1). Our results reiterate the assertion that genes/QTL for ‘domestication’ traits often correspond across divergent lineages (Paterson *et al.* 1995) providing a leverage to accelerate genetic gains in predictive breeding of marginalized grasses like bermudagrass.

### Implications for bermudagrass breeding

We found significant genotypic effects (*P* < 0.001) and positive correlations among the morphological traits, consistent with a prior study in bermudagrass (Wu *et al.* 2007). Exploratory analyses produced four key observations of interest in bermudagrass breeding: first, epistatic interactions were important and mostly involved M-QTL; second, QTLx*E* interactions were scarce and of comparatively lower magnitudes; third, a number of QTL underlying the traits of interest were colocalized; and fourth, both parents contributed QTL with contrasting phenotypic effects.

Epistatic interactions are important components of trait variation including plant morphology in grasses (Guo and Hong 2010; Ku *et al.* 2012; Wang *et al.* 2012; Yang *et al.* 2016). In the current study, significant epistatic interactions (*R*^2^αα = 1.1–22.5%) were detected by MCIM for each of the five traits in separate and/or joint environment analyses. Most epistatic interactions (64%) identified by GMM involved at least one significant S-QTL. In fact, a number of S-QTL (30) detected at a stringent significance threshold (avg. *P =* 0.001) with high main effect contribution to the traits (avg. *R*^2^ = 13%) were also involved in epistatic interactions. Further, 9 of 16 *αα* interactions identified by MCIM had at least one M-QTL. Interestingly, two of the int-QTL involved digenic interactions between previously detected pairs of M-QTL. For example, CIM detected significant main effects for QTL qILEN-6-1 and qILEN-5-1 for stolon internode length and qLW-3-1 and qLW-3-2 for leaf width, while their interactions were also significant in both separate and joint environment analysis following MCIM. On the other hand, QTLx*E* interactions were largely absent - a few inconsistent cases contributed little (*R*^2^αe = 0.7–1.2%; *R*^2^ααe ∼2%) to overall trait variances (Table 3), indicating that detected QTL are mostly ‘stable’. Besides, colocalization of QTL (Table 2; Supplemental File 2, Table S2.4) suggests that the genetic basis of stolon and foliage morphology (except that between LLEN and LW, and between LLS and leaf traits) could be determined by pleiotropic or tightly linked genes, indicating the feasibility of simultaneous improvement of the target traits by breeding. QTL correspondence with allied grasses showed that several orthologous/syntenic regions harbor plant morphology QTL, not only those of interest in the current study (Supplemental File 2, Table S2.2), but also involving seedhead-related characteristics (data not shown). However, pleiotropy *vs.* tight linkage and conservation of orthologous function in divergent lineages are issues that may be clarified by fine mapping and characterization of causative allelic variants at these loci. Further, the parental species of the current study contributed alleles that confer contrasting phenotypic effects in the hybrid population, which indicates that both carry favorable alleles suited for alternative breeding goals (*i.e.*, turf or forage breeding).

Phenotypic recurrent selection has been successful in improving turf morphological traits in *C. dactylon* var. *dactylon* breeding for seeded bermudagrass cultivars (Taliaferro 2003). QTL analyses suggest that molecular breeding approaches like marker-assisted or genomic selection are feasible. It is in this context that we would like to reiterate that our mapping population is an F_1_ interspecific triploid, a reproductively sterile population that cannot be subjected to population breeding approaches. Nevertheless, selection of parents or their hybrids, hitherto exclusively dependent upon phenotypic selection, can be aided by marker-assisted selection (MAS). In intraspecific *C. transvaalensis* populations studied in greenhouse environments, genetic variance of plant height, internode length, stolon length, and leaf length constituted both additive and dominance components (the latter contributing more to the total), suggesting that clonal selection of F_1_ hybrids is desirable for improving the traits under consideration (Kenworthy *et al.* 2006). Owing to the limitation of pseudo-testcross mapping with a limited number of allelic biparental makers, we could not partition QTL effects into additive or dominance components in the present study. The QTL identified in this study, especially qILEN-3-2 (for ILEN), qLLS-7-1 (for LLS), qLEN-1-1 (for LLEN), and qLW-3-2 (for LW) represent potential candidates for fine mapping and for further validation in bermudagrass breeding populations. QTL correspondence between bermudagrass and other grasses suggests that ‘predictive breeding’ may be a fruitful avenue for improving traits of interest in bermudagrass.

### Conclusion

In the present study, we dissected the genetic basis of complex morphological traits of interest in turf bermudagrass. A number of genomic regions with significant contributions to the traits were detected, which can be targeted in fine mapping and introgressive breeding. This early QTL study in bermudagrass adds to molecular breeding resources and information in the genus. There is much potential for advancing current research by increasing the size of the mapping population, increasing marker densities using state-of-the-art genomic tools, integrating parental linkage maps using biparental bridge markers, and collecting more extensive phenotype data (*i.e.*, multi-environment across several locations over multiple years). Turfgrass performance (*i.e.*, establishment and recovery speed, canopy density and uniformity after mowing, and other aesthetic properties) also varies with different management strategies (*i.e.*, mowing height, mowing frequency, fertilizer input, growth-regulator application) and different environmental conditions. Therefore, QTL studies under different management plans and/or growth environments are also warranted.
